# Phylogenomic associations among methicillin-resistant *Staphylococcus aureus* isolates derived from pets, dairies, and humans

**DOI:** 10.1128/spectrum.01995-24

**Published:** 2025-04-30

**Authors:** Margaret Krueger, Shayla Bajric, Sandra Godden, Jeffrey B. Bender, Rinosh Mani, Srinand Sreevatsan

**Affiliations:** 1 Department of Pathobiology and Diagnostic Investigation, College of Veterinary Medicine, Michigan State University, East Lansing, Michigan, USA; 2 Department of Veterinary Population Medicine, College of Veterinary Medicine, University of Minnesota, Minneapolis, Minnesota, USA; 3 Bacteriology/Mycology Division, Veterinary Diagnostic Laboratory, Michigan State University, East Lansing, Michigan, USA; 4 College of Veterinary Medicine, University of Missouri, Columbia, Missouri, USA; Universidade Federal do Rio de Janeiro, Rio de Janeiro, Brazil

**Keywords:** *Staphylococcus*, pets, MRSA, phylogenomics, One Health, interspecies transmission

## Abstract

Methicillin resistance in *Staphylococcus aureus* is conferred by the mobile genetic element, staphylococcal cassette chromosome *mec* (SCC*mec*). Methicillin-resistant *Staphylococcus aureus* (MRSA) can transmit among animals and humans, leading to persistence and back transmission events. The current study tested the hypothesis that companion animal and livestock-associated (LA) MRSA isolates share genomic similarity, suggesting shared ancestry with hospital-associated (HA) or community-associated (CA) MRSA. Eight *S*. *aureus* isolates from therapy dogs (*n* = 5) and bulk tank milk (*n* = 3) were genome sequenced, and 71,721 genome-wide single nucleotide polymorphism (SNP) locations were extracted and phylogenetically compared against methicillin-sensitive *Staphylococcus aureus* (MSSA) and MRSA genomes of isolates from a variety of species and time frames, available in the National Center for Biotechnology Information (NCBI) database. A maximum likelihood phylogenetic tree was constructed to define *S. aureus* lineages across isolates from animals and humans. Four isolates from companion animals and three bulk tank milk isolates clustered with human isolates, while one companion animal isolate clustered with genomes of MRSA isolated from swine. Four therapy dog isolates had CA-MRSA SCC*mec* types IVa, IVc, and V/VII, respectively, while one therapy dog and one bulk tank milk isolate shared SCC*mec* type (IIa) that is commonly seen in HA-MRSA. Two isolates from bulk tank milk were methicillin sensitive and did not carry *mecA*.

**IMPORTANCE:**

Methicillin-resistant *Staphylococcus aureus* (MRSA) infections are a major medical concern, causing a range of conditions from skin infections and invasive disease to death. MRSA was discovered as a nosocomial infection; however, it has since been isolated in communities and animals worldwide. This research was significant because canine and bulk tank milk isolates were found to have genomic relatedness to human and domestic animal *S. aureus* isolates. This genetic relatedness implies either a parallel evolution within hosts converging to successful genotypes or real interspecies transmission events among animals and humans.

## INTRODUCTION

*Staphylococcus aureus* colonizes the skin and nasal tract of animals and humans (1–3). This bacterium may pass back and forth between human and animal hosts harmlessly until the right opportunity presents, such as contact with broken skin or an immunocompromised host, leading to an infection (4–6). *S. aureus* with methicillin resistance (MRSA) was first detected in 1961 and has since been reported worldwide, including animal species such as pigs, horses, cows, and companion animals (7–9).

*S. aureus* colonization has been reported in 20% to 40% of human nasal tracts (3, 10, 11), where 1.5% of the US population carries methicillin-resistant *S. aureus* (MRSA) (12). Whereas isolates collected from dogs during veterinary visits had *S. aureus* colonization at 8% with 2.7%–4% being MRSA (1, 2, 13), *S. aureus* is also prevalent on the farm, with 77% of pigs testing positive for *S. aureus* and 9% MRSA prevalence (14). While 40% of bulk tank milk had *S. aureus*, MRSA prevalence ranges between 0% and 4% (15, 16).

When humans interact with animals, professionally, in households, or as therapy dogs, there is a possibility of bacterial transmission and colonization, which can serve as a mechanical vector, leading to new infections or back transmission events (17–21). MRSA infections are a major medical concern, causing a range of conditions from skin infections to invasive diseases including bacteremia, necrotizing fasciitis, endocarditis, abscesses, osteomyelitis, and pneumonia (2, 22, 23). The MRSA economic burden in the United States is $478 million annually for a community-associated MRSA infection when outpatient, emergency room/hospital visits, treatment, mortality, and work absenteeism costs are considered (24). On the farm, MRSA can be costly also, with it being estimated it would cost $2.37–$2.58 billion to eradicate Livestock-associated (LA)-MRSA from pig housing units in Denmark (25). The economic burden from companion animal MRSA infections is not fully understood but could cost individual animal guardians less than $100 or thousands in veterinary costs, depending on whether antibiotic treatment or surgery is needed.

This study hypothesized that MRSA isolated from therapy dogs and bulk tank milk is genomically related to MRSA associated with human infections derived from either hospital or community origins. Furthermore, genetic similarity to MRSA isolated from felines, bovines, swine, and canines was investigated.

## RESULTS

### Reconfirming *S. aureus* bacterial species and phenotypic properties

The 10 isolates collected from therapy dogs (TD1, TD2A, TD3, TD4, TD5, and TD82) or bulk milk tanks (FA6, FA20, FA25, and SP12) species were identified with duplicate matrix-assisted laser desorption ionization-time of flight (MALDI-ToF) analyses (Table 1). Eight isolates were reconfirmed as *Staphylococcus aureus*, while samples TD2A and FA25 were identified as *Staphylococcus pseudintermedius* and *Enterococcus faecalis*, respectively, and therefore excluded from genome sequencing. The other eight isolates were whole genome sequenced (WGS) and identified as 91%–97% *S. aureus* by Kraken2, confirming the MALDI-ToF results (Table 1).

**TABLE 1:** *S. aureus* confirmation and SCC*mec* classification

Sample	Organism identification	MALDI-ToF score^*a*^ (analysis 1/2)	% *S. aureus* identification with Kraken2	Kirby Bauer disk diffusion zone of inhibition (mm)^*b*^	Coagulase result^*c*^	Multiplex PCR SCC*mec* type	SCC*mec* type Staphopia-sccmec
MALDI-ToF control	*E. coli*	2.17/2.09	N/A^*d*^	N/A	N/A	N/A	N/A
TD1	*S. aureus*	2.31/2.15	92.8	15 (R)	+	IVa	IVa
TD2A	*S. pseudintermedius*	1.92/1.70	N/A	N/A	N/A	Not present	N/A
TD3	*S. aureus*	2.37/2.23	94.5	14 (R)	+	Inconclusive	IVc
TD4	*S. aureus*	2.27/2.23	95.5	6 (R)	+	II	IIa
TD5	*S. aureus*	2.3/1.82	91.3	14 (R)	+	IVa	IVa
TD82	*S. aureus*	2.33/2.23	91.1	15 (R)	+	V	V/ VII
Sp12	*S. aureus*	2.31/2.23	95.3	6 (R)	+	II	IIa
Fa6	*S. aureus*	2.28/2.19	96.5	26 (S)	+	Not present	No *mecA*
Fa20	*S. aureus*	2.31/2.03	96.8	26 (S)	+	Not present	No *mecA*
Fa25	*E. faecalis*	2.06/2.05	N/A	N/A	N/A	Not present	N/A

^*a*^ MALDI-ToF scores of 0.00–1.69 signify no reliable identification; scores of 1.70–1.99 identify to the genus level; and scores of 2.00–3.00 reliably identify organism to the genus and species levels.

^*b*^ Kirby Bauer disk diffusion results using 6 mm cefoxitin antibiotic disks for *S. aureus*. Greater than or equal to 22 mm indicates antibiotic susceptibility (S), while ≤21 mm indicates antibiotic resistance (R).

^*c*^ Isolates were considered coagulase positive (+) if there was evidence of clotting when emulsified with rabbit coagulase plasma. If there was no evidence of clotting after 24 hours, the isolate was considered coagulase negative (–). Multiplex PCR was developed by Zhang et al. (26).

^*d*^ N/A = Not available or not performed.

*Staphylococcus aureus* with methicillin and other beta-lactam resistances is a major concern in medical communities; therefore, eight isolates identified as *S. aureus* were tested for methicillin resistance using cefoxitin (Table 1). Five canine and one bulk tank milk *S. aureus* isolates (TD1, TD3, TD4, TD5, TD82, and SP12, respectively) were resistant to cefoxitin, while two bulk tank milk isolates were susceptible (FA6 and FA20). A characteristic of *S. aureus* is coagulase production that can distinguish them from most other *Staphylococcus* species (27). All eight *S*. *aureus* isolates showed evidence of clotting within the 2-hour time point (Table 1). These phenotypic tests confirm the eight isolates are *S. aureus* and six are methicillin resistant.

### HA- and CA-SCC*mec* types

**Fig 1:**
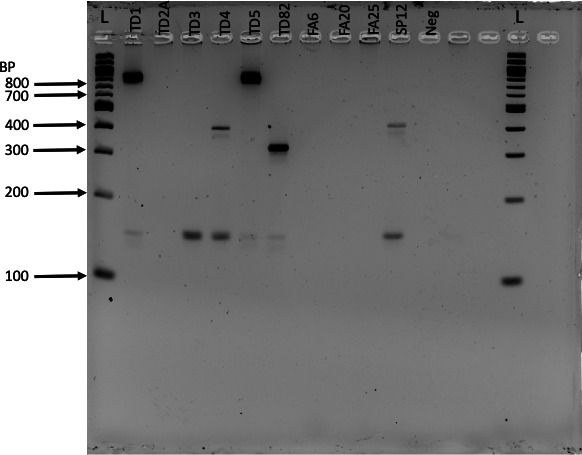
*S. aureus* multiplex PCR to find SCC*mec* types using primers designed by Zhang et al. (26). The ladder (L) was a 100 base pair (bp) marker. Isolates TD1 and TD5 are SCC*mec* type IVa (fragment size 776 bp). Isolates TD4 and SP12 are SCC*mec* type II (fragment size 398 bp). Isolate TD82 is SCC*mec* type V (fragment size 325 bp). Isolate TD3’s SCC*mec* type was undetermined. *mecA* was present in isolates TD1, TD3, TD4, TD5, TD82, and SP12 (fragment size 147 bp). Isolates TD2A, FA6, FA20, and FA25 did not carry the *mecA* type.

To define if our isolates had hospital-associated (HA-MRSA) or community-associated (CA-MRSA) MRSA staphylococcal cassette chromosome *mec* (SCC*mec)* types, a multiplex PCR developed by Zhang et al. (26) was used. The method determined *S. aureus* isolates TD1 and TD5 were SCC*mec* type IVa, isolate TD82 was SCC*mec* type V, and isolates TD4 and SP12 were SCC*mec* type II, which was consistent with Haran et al.’s (16) findings for SP12. Isolate TD3 was non-typable with the multiplex PCR method. There was no distinct 147 base pair band for this isolate except for *mecA*, which was present in all the MRSA isolates (Fig. 1). Lastly, isolates TD2A, FA6, FA20, and FA25 were multiplex PCR negative. For isolates FA6 and FA20, the absence of SCC*mec* implies methicillin sensitivity, as has been established (16).

Using the Staphopia-sccmec software, the FA6 and FA20 isolates were classified as false for *mecA* presence, implying they are methicillin-sensitive *S. aureus* (MSSA), which was confirmed by the disk diffusion analysis and multiplex PCR. Four isolates (TD1, TD3, TD5, and TD82) carried the CA-MRSA SCC*mec* types (IVa, IVc, IVa, and V/VII, respectively), while isolates TD4 and SP12 carried the HA-MRSA SCC*mec* types (IIa) (Table 1). This finding is consistent with the multiplex PCR results, except the *in silico* work was able to classify isolate TD3’s SCC*mec* type and revealed that isolate TD82 had multiple SCC*mec* cassettes.

### Single nucleotide polymorphisms (SNPs) to find isolate relatedness

**Fig 2:**
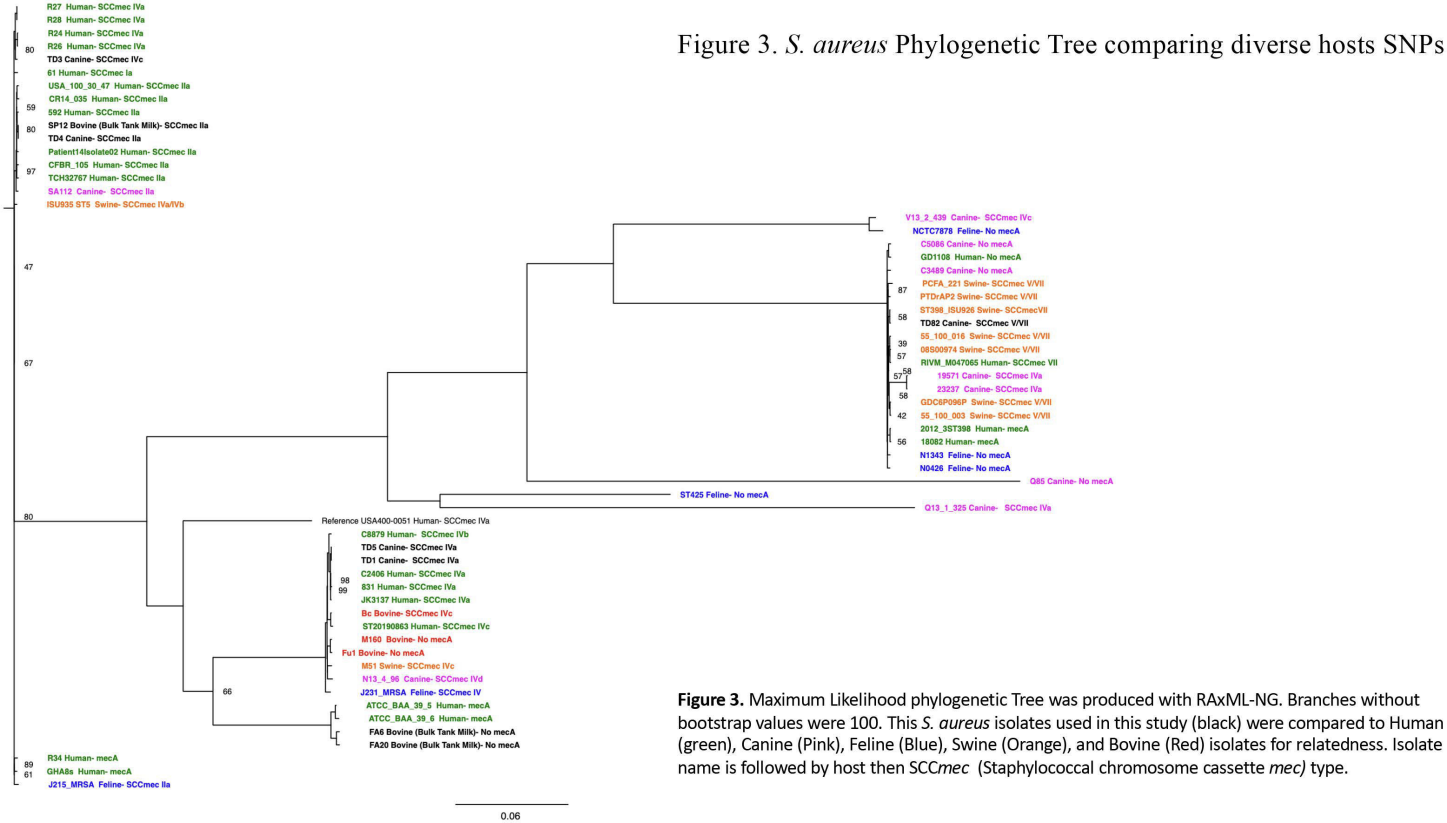
Maximum likelihood phylogenetic tree constructed using RAxML-NG shows a genome-level relatedness of animal and human MRSA isolates. Branches without bootstrap values were 100. The *S. aureus* isolates used in this study (black) were compared to human (green), canine (pink), feline (blue), swine (orange), and bovine (red) isolates for relatedness. Isolate name is followed by host, then SCC*mec* type.

To find isolate relatedness, 71,721 SNP locations from the eight *S*. *aureus* isolates and 52 publicly available *S. aureus* genomes were phylogenetically compared (Fig. 2). The animal MRSA isolate genomes analyzed in the present study clustered in the clade with either human (TD1, TD3, TD4, TD5, TD82, and SP12), canine (TD1, TD3, TD4, TD5, TD82, and SP12), swine (TD1, TD3, TD4, TD5, TD82, and SP12), feline (TD1, TD5, and TD82), or bovine (TD1 and TD5) *S. aureus* SNP groups. The methicillin-sensitive isolates FA6 and FA20 clustered with human MRSA SNP groups.

### SNPs aligned on the genome

**Fig 3:**
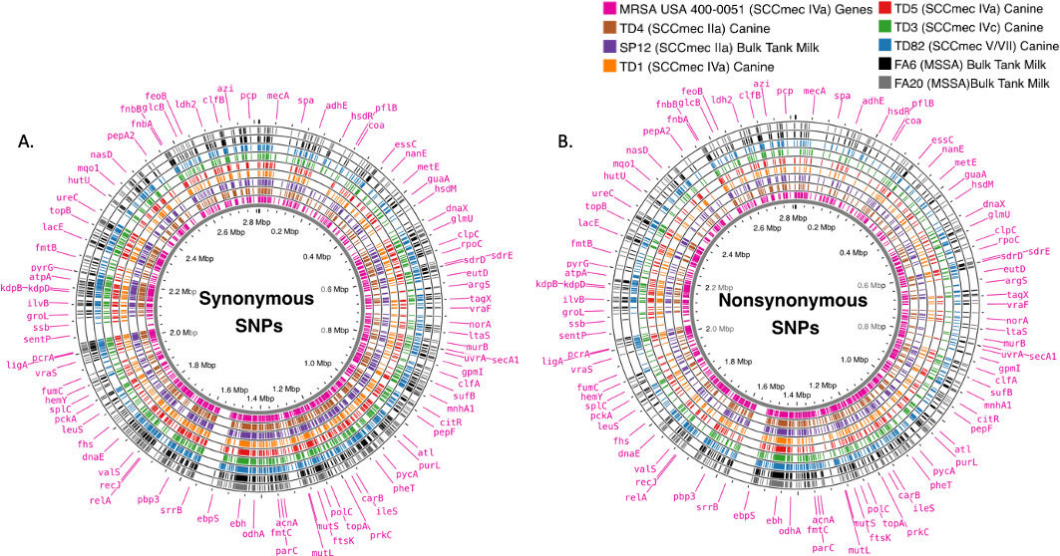
The genes of MRSA USA 400–0051 (reference strain) with putative functions: pink, ring 1 (innermost), is plotted with isolates TD4 (brown, ring 2), SP12 (purple, ring 3), TD1 (orange, ring 4), TD5 (red, ring 5), TD3 (green, ring 6), TD82 (blue, ring 7), FA6 (black, ring 8), and FA20 (gray, ring 9 [outermost]) synonymous (**A**) and nonsynonymous (**B**) SNPs in consecutive circles. The inner circle is the isolate genome size in million base pairs (mbp). The Circos map was generated using Proksee, which utilized Prokka’s annotation data.

Genome-wide SNP distribution for each genome was plotted on Circos maps (Fig. 3) to show no genomic regional bias. Specific genome and SNP statistics are in Table 2. The genome sizes of the isolates ranged from 2,721,527 to 2,922,075 base pairs. The coding sequences (CDS) have between 2,465 and 2,747 open reading frames. There were between 504 and 709 genes with synonymous SNPs, while the isolates had between 318 and 488 genes with nonsynonymous SNPs. The reference genome had 823 genes with putative functions, comparatively, each of our isolates had hundreds of genes with SNPs, where isolate TD82 had the most; 86% and 59% of genes had synonymous and nonsynonymous SNPs, respectively.

**TABLE 2:** Single nucleotide polymorphisms identified by isolate

Isolate	Total genome length (bp)^*a*^	Coding sequences (CDS)^*a*^	Genes with synonymous SNPs^*b*^	Genes with nonsynonymous SNPs^*b*^	% Genes with synonymous SNPs^*c*^	% Genes with nonsynonymous SNPs^*c*^
TD1	2,922,075	2747	504	318	61	39
TD3	2,783,259	2563	578	363	70	44
TD4	2,721,609	2465	574	371	70	45
TD5	2,920,586	2742	513	320	62	39
TD82	2,827,164	2618	709	488	86	59
FA6	2,743,801	2533	549	373	67	45
FA20	2,774,896	2578	549	372	67	45
SP12	2,721,527	2466	622	371	76	45

^*a*^ Determined by Prokka.

^*b*^ Genes with putative functions determined by Snippy.

^*c*^ Based on reference MRSA 400-0051, 823 annotated genes with putative functions.

### Antimicrobial agent resistance genes

Six of the isolates used in this study (TD1, TD3, TD4, TD5, TD82, and SP12) had *mecA* and tested positive for cefoxitin resistance. As antimicrobial resistance can increase the severity of bacterial infections, the canine and bulk tank isolates from this study were analyzed for other antimicrobial agent resistance (AMR)-associated genes (28). All isolates carried multiple AMR genes except isolates FA6 and FA20, which did not have any, with the exception of tet (29) (Table 3). This gene (tet) encodes a tetracycline efflux pump, which was also present in all isolates and in the publicly available complete genomes (data not shown) (30). Isolate TD82 clustered closest to swine isolates and carried tetracycline-resistant genes tet (M) and tet (K), which has a high prevalence among LA-MRSA (31). The animal-derived MRSA isolates had antibiotic resistance genes for methicillin and beta-lactams, along with other antibiotic resistance-associated genes, including bleomycin (in canine isolates TD4 and TD82 and a bulk tank isolate SP12), macrolides (TD1, TD5, TD3, TD4, TD82, and SP12), streptomycin (TD1 and TD5), and fosfomycin (TD1, TD5, TD3, TD4, and SP12).

**TABLE 3:** Antimicrobial resistance genes of *S. aureus* isolates in the present study^*a*^

Isolate (SCC*mec* type)									
Gene	Resistance conferred	TD1 (IVa)	TD5 (IVa)	TD3 (IVc)	TD4 (IIa)	SP12 (IIa)	TD82 (V/VII)	FA6 (MSSA)	FA20 (MSSA)
*mecI_of_mecA*	Methicillin								
*mecR1*									
*mecA*									
*blaR1*	Beta-lactam								
*blaI_of_Z*									
*blaZ*									
*blaPC1*									
*tet (38*)	Tetracycline								
*tet(M*)									
*tet(K*)									
*msr(A*)	Macrolide								
*mph(C*)									
*erm(C*)									
*erm(A*)									
*aph(2'')-Ih*	Amikacin, gentamicin, kanamycin, tobramycin								
*vga(A*)	Lincosamide								
*fosB-Saur*	Fosfomycin								
*sat4*	Streptothricin								
*bleO*	Bleomycin								
*ant (9)-Ia*	Spectinomycin								
*aph(3')-IIIa*	Amikacin, kanamycin								
*ant(4')-Ia*	Kanamycin, tobramycin								

^*a*^ Gray shading represents the antibiotic resistance genes present in those specific isolates.

## DISCUSSION

The objective of this study was to determine if methicillin-resistant and susceptible *S. aureus* isolates collected from therapy canines and bulk tank milk in Minnesota dairies shared genome-level relatedness with MRSA isolated from multiple animal species. *S. aureus*’s SCC*mec* transposon is categorized into two major groups, hospital (HA) or community (CA) associated, which can affect how an MRSA infection presents. CA-MRSA contains smaller SCC*mec* elements (types IV and V) and typically causes skin and soft tissue infections. HA-MRSA is typically more invasive, contains larger SCC*mec* types (I, II, and III), and is associated with infections originating from health care settings (32–35). Even though CA- and HA-MRSA SCC*mec* types can be used to determine MRSA strain origins, they are not reliable to determine an infection’s origin because both CA- and HA-MRSA are currently in hospitals and long-term care facilities (36–38). Therapy dogs interact with hospital and community environments; therefore, our isolates having both CA- and HA-SCC*mec* types was expected. However, when comparing the therapy dog isolates to genomes from multiple species, we found genetic similarity to human, bovine, feline, swine, and canine isolates, implying that our therapy dog MRSA isolates share a common ancestor with isolates collected from multiple species and spread through interspecies transmission. When examining the bulk tank milk isolates, two were MSSA but clustered closely with human isolates, while MRSA isolate SP12 had an HA-SCC*mec* type (IIa) and clustered with human, canine, and swine isolates. This implies SP12’s genomic ancestry is from a hospital setting that has been passed to the farm setting, and all three bulk tank milk isolate infections are the results of interspecies transmission.

This study’s canine isolates were from therapy dogs, which have an increased risk of acquiring and/or spreading interspecies infections in healthcare settings. However, most companion animals do not enter these facilities; therefore, the risk attribution for healthcare facility interspecies transmission is low compared to animal interactions with humans in the community. Certain occupations such as veterinarians (companion and production animal specialties), farm employees, or healthcare workers who come in close contact with animals or patients are more likely to become carriers or vectors of MRSA, increasing the risk of MRSA transmission to companion animals during interactions (17–21). Previous studies have shown evidence of *S. aureus* interspecies transmission where isolates collected from humans and their companion animals are genotypically indistinguishable or having the same *Staphylococcus aureus* Protein A (SPA) type (21, 29, 39, 40). When an animal is infected with MRSA, there can be profound consequences. Besides becoming a vector that can spread MRSA, the animal will need treatment, which can range from antibiotic use to euthanasia. It should be noted that animals are often the interspecies transmission recipient because MRSA can be spread by human interactions and environmental contamination (4, 41). According to the Centers for Disease Control and Prevention (https://www.cdc.gov/mrsa/about/index.html), MRSA is often spread among humans from contact with contaminated hands or wounds, which may also be a source of infection or nasal/skin contamination and carriage among companion animals. Practicing proper hygiene with companion and production animals, such as hand washing and cleaning surfaces, may prevent them from becoming MRSA vectors and/or reduce back transmission events (42).

For this study, multiple methods were used to confirm the identity of our isolates (multiplex PCR, MALDI-ToF, and *in silico*). Most methods supported each other; however, isolates TD3 and TD5’s SCC*mec* type identification could not be solely accomplished by a multiplex PCR. Isolate TD3 was untypable with the multiplex PCR, but the genotype was determined to be SCC*mec* type IVa *in silico*. The inability to type the SCC*mec* among some MRSA isolates is likely explained by multiple reasons, including undiscovered/uncommon SCC*mec* types or PCR or software limitations. The inability to find MRSA’s SCC*mec* type has been previously reported by Nagasundaram and Sistla (43) and Wang et al. (31), noting 1.5%, 7.5%, and 87% of MRSA isolates as non-typable. While the one canine isolate TD82 was classified as SCC*mec* type V by the multiplex PCR, genomic analysis revealed that it carried multiple SCC*mec* types V and VII. These two SCC*mec* types differ by the *mec* gene complex structure; the IS431 upstream of *mecA* is in the opposite orientation of *mecA* for SCC*mec* type V, while SCC*mec* type VII has the upstream IS431 in the same orientation as *mecA* (35). Even though these SCC*mec* types suggest similarity within subtype, they are thought to have evolved independently (44, 45), implying that the SCC*mec* complex may be introduced into the bacterial genome during separate horizontal gene transfer events. Previously, multiple SCC*mec* types have been reported in 1.1% to 74% of MRSA isolates (26, 36, 43). While seven of the publicly available genomes used in this study had multiple SCC*mec* types, all were isolated from swine (Table S1). Having multiple SCC*mec* types could be advantageous to MRSA because SCC*mec* may carry genes that help *S. aureus* survive in diverse environments, in addition to other antibiotic resistance-associated genes (44).

Bacteria with antimicrobial resistance genes, such as MRSA, can increase infection complications leading to longer infection times, increased hospital stays, and increased risk of complications including death (28). Multiple drug resistance (MDR) genes increase the risk of complications even more. Examining the AMR genes present in MRSA isolates gives a better understanding of how to overcome MRSA infections. All of the MRSA isolates in this study (TD1, TD3, TD4, TD5, TD82, and SP12) carried multiple AMR genes. This confirms when Haran et al. (16) phenotypically assessed bulk tank milk isolate SP12 for antibiotic susceptibility and showed that the isolates were resistant to 14 out of 22 antibiotics, including cefoxitin. Haran et al. (16) also reported four other bulk tank milk isolates as having at least three MDR genes and identified isolates FA6 and FA20 as MSSA, which agrees with this study’s findings. MDR resistance has also been found in MRSA-positive nasal swabs of healthy farm animals (sheep, goats, cattle, and buffalo), with all having resistance to at least four antibiotics (46). Penna et al. (2) found canine MRSA isolates had multidrug resistance, while Davis et al. (1) reported genes erm(A), blaZ, and *mecA* present in SCC*mec* type II isolates, which was similar to isolates TD4 and SP12. MDR MRSA has been reported in human and canine hosts, with Bhutia et al. (36) reporting 33% of human MRSA isolates having the MDR phenotype. In a study by Tomlin et al. (47) involving 11 dogs with MRSA, all were found to be resistant to oxacillin and methicillin, where eight of the dogs had up to four additional antimicrobial resistances among those tested.

Often, MRSA infections are treated with vancomycin; however, vancomycin-resistant MRSA isolates have been reported (48, 49). János et al. (49) reported vancomycin resistance and other MRSA first-line antibiotics imipenem and rifampicin in *S. pseudintermedius* canine isolates, a bacterium that is often misidentified as *S. aureus* in humans (50). Co-infection with *S. aureus* and *S. pseudintermedius* could lead to increased antibiotic resistance in MRSA through horizontal gene transfer with other *Staphylococcus* spp., which could further complicate MRSA treatment (49–52).

Traditionally, genomic relatedness for *S. aureus* is found through multilocus sequence typing (MLST) or SPA typing. However, both MLST and SPA typing use a limited number of genes (seven for MLST and one for SPA typing) (53). The seven MLST genes range from 402 to 516 bp, giving a theoretical maximum of 3,198 SNP locations, but the actual polymorphic nucleotide sites are likely much smaller (54). While the *spa* gene has tandem repeats in the X region causing variation in gene size, Shakeri et al. (55) reported amplicon sizes ranging between 1,150 and 1,500 bp. By focusing on genome-wide SNPs, 71,721 SNP locations were compared to find relatedness, giving a deep comparison of genomes because this study’s SNP location counts are 22- and 48-fold larger compared to the maximum theoretically possible locations for MLST and SPA typing, respectively. Figure 3 shows the gene distribution of the reference strain (MRSA 400-0051) and which genes this study’s isolates had synonymous and nonsynonymous SNPs (individual isolate’s gene and SNP statistics are found in Table 2).

Using genome-wide SNPs, the *S. aureus* isolates in this study were found to have genomic relatedness to human, canine, feline, swine, and cattle isolates. Clustering with multiple host isolates suggests sharing a common ancestor and implies interspecies transmission of MRSA.

## MATERIALS AND METHODS

### Bacterial isolates

Ten archived *Staphylococcus* isolates from companion animals and bulk tank milk were revived from frozen glycerol stocks onto Brain Heart Infusion (BHI) agar (BD, catalog number 211059) and were incubated at 37°C for 18 hours. Single colonies were either sub-cultured in 10 mL BHI broth or BHI agar plates, with incubation under the same conditions. Four of the isolates (FA6, FA20, FA25, and SP12) were originally collected in 2009 from pooled bulk tank milk (16). The remaining isolates were nasal and wound swabs from therapy dogs (TD1, TD2A, TD3, TD4, TD5, and TD82) collected in 2009 (56). After collection, all isolates were typed at the Minnesota Veterinary Diagnostic Laboratory (VDL) and were stored at −80°C.

### MALDI-ToF and Kirby Bauer disk diffusion analysis

Less than 24-hour-old single colonies were species identified using MALDI-ToF (Bruker Microflex, USA) at Michigan State University’s VDL. Once bacterial identities were reconfirmed as *Staphylococcus* spp., antimicrobial resistance profiles were analyzed using the Kirby Bauer disk diffusion method. Two to three colonies were added to a saline solution, calibrated to 0.5 McFarland units, and streaked onto 2% NaCl Mueller Hinton agar plates (Thermo Scientific, catalog number R01621) with a 6 mm cefoxitin disk (Hardy Diagnostics, catalog number Z8241). The plates were incubated at 37°C for 18 hours, and results were interpreted according to the Clinical and Laboratory Standards Institute (CLSI) guidelines. If the zone of inhibition diameter was greater than or equal to 22 mm, the isolate was deemed susceptible to the antibiotic, and if less than or equal to 21 mm, it was considered resistant.

### Coagulase test to confirm *S. aureus*

The eight frozen stock isolates identified by MALDI-ToF as *S. aureus* were further confirmed using the tube coagulase method. In brief, 0.5 mL reconstituted rabbit coagulase plasma (BD, catalog number 240827) was pipetted into a test tube, and two to three fresh bacterial colonies were collected from BHI agar plates with a sterile inoculating loop and were emulsified in the plasma. The tube was incubated at 37°C for 4 hours and examined for evidence of clotting at 2, 3, and 4 hours. Next, the tubes were placed at room temperature overnight for a final observation at 24 hours post-inoculation. Evidence of clotting indicated the organism was coagulase positive. An *S. aureus* isolate confirmed with MALDI-ToF was used as a positive control, and nuclease-free water was used as the negative control.

### Bacterial DNA extraction

Total bacterial DNA was extracted using the Qiagen DNeasy Blood & Tissue Kit (Qiagen, Redwood City, CA, USA). An additional bacterial pellet wash step with 1.5 mL phosphate-buffered saline (PBS) (Fisher Scientific, catalog number BP3994) was included to remove remnant media and optimize DNA quality. The Zymo Research DNA Clean and Concentrator-10 kit (Zymo Research, catalog number D4011) was used to concentrate DNA samples to meet sequencing parameters.

### Multiplex PCR for SCC*mec* subtyping

A multiplex polymerase chain reaction (PCR) assay modeled after Zhang et al. (26) was used to determine the SCC*mec* element types present in the MRSA-positive samples. The PCR used the types I, II, III, IVa, IVb, IVc, IVd, V, and *MecA*147 forward and reverse primers at the concentrations published by Zhang et al. (26). The reaction contained 1× PCR buffer (1.5 mM MgCl_2_) concentration, 200 µM of each dNTP (dATP, dCTP, dGTP, and dTTP), 2.5 units HotStarTaq, and 2 µL isolate template DNA (about 50–100 ng per reaction). The reaction was brought to 50 µL with nuclease-free water (Qiagen catalog number 129114). The HotStarTaq master kit (Qiagen catalog number 203445) provided the PCR buffer and HotStarTaq, while the dNTPs were purchased from New England Biolabs (catalog number N0447L).

The thermal cycler setting was 95°C for 15 minutes followed by 35 cycles of 94°C for 30 seconds, 55°C for 30 seconds, and 72°C for 30 seconds. After the 35 cycles, the samples were held at 72°C for 5 minutes then stored at 4°C. The amplicons were visualized by running in 3.5% agarose gel (Corning, catalog number ARG-LE-500) in 1× TAE buffer with 0.5 ng/mL ethidium bromide. The gel was electrophoresed at 100 V for 100 minutes using a 100 base pair ladder (New England Biolabs, catalog number N3231S) and was visualized on the ChemiDoc Imaging System (Bio-Rad, USA).

### Whole genome sequencing and genome assembly

Whole genome sequencing of the eight *S*. *aureus* isolates was performed on the NovaSeq PE150 Illumina sequencing strategy and NovaSeq 6000S4 sequencing platform using the Nextera XT DNA library preparation kit (Novogene, Sacramento, CA, USA). The raw reads were uploaded to the Bacterial and Viral Bioinformatics Resource Center (BV-BRC, https://www.bv-brc.org/) to check for contamination using Kraken2 (57) with default settings (Taxonomic Classification). Using the Fastq Utilities, the reads were trimmed using Trim Galore version 0.6.5dev (developed by Felix Krueger at the Babraham Institute, https://github.com/FelixKrueger/TrimGalore) and Cutadapt version 2.2 (58). The trimmed reads were then quality checked with FastQC version 0.11.9 (developed by Simon Andrews at the Babraham Institute, https://github.com/s-andrews/FastQC).

The trimmed reads were downloaded from BV-BRC and uploaded to Michigan State University’s High Performance Computing Cluster (HPCC) for *de novo* assembly into contigs using Shovill 1.1.0 (https://github.com/tseemann/shovill) with default settings, except the minimum contig length was set at 300 base pairs. The assembled contigs were corrected and scaffolded using RagTag v2.1.0 (59, 60). The quality of the contigs or draft genomes was assessed using Quast 5.0.0 (https://github.com/ablab/quast). All software programs were used with default settings unless noted otherwise. The MRSA strain USA 400-0051 (GenBank Accession CP019574.1) was used as the reference genome for the RagTag and Quast analysis.

### Single nucleotide polymorphisms

Genome-wide SNPs were inferred using Snippy v4.6.0 (https://github.com/tseemann/snippy) with default settings, except snpEFF version 5 was reverted to version 4. Eight isolates (TD1, TD3, TD4, TD5, TD82, FA6, FA20, and SP12) and publicly available genomes were analyzed in batch by utilizing Snippy-multi with USA 400-0051 as the reference genome. MRSA 400 is a well-characterized human MRSA strain to compare our isolates to find interspecies genomic relatedness (61, 62). Snippy-core’s output (.aln) was used to build phylogenetic trees. Annotated genes with putative functions that had SNPs were plotted on a Circos map using Proksee (63). Proskee utilized genome annotation data generated using Prokka v 1.14.6 at beginner settings (64). Prokka uses NCBI BLAST+ (65), ISfinder (https://isfinder.biotoul.fr/), NCBI Bacterial Antimicrobial Resistance Reference Gene Database (https://www.ncbi.nlm.nih.gov/bioproject/313047), and UniProt (https://www.uniprot.org/) to assign putative functions to the CDS.

### *S. aureus* genome database and phylogenetic analysis

Thirty-eight complete and 14 draft publicly available *S. aureus* genomes isolated from North and South America, Europe, Africa, Australia, and Asia were included for phylogenetic analysis. In a preliminary study, 170 genomes were compared to our isolates, and the 52 most relevant genomes were selected for this study. Canine and feline host genomes were expanded to include draft genomes because the BV-BRC website only had one complete genome for each species. The publicly available *S. aureus* genomes used in this study were derived from human, feline, bovine, canine, and swine hosts, with collection dates ranging from 1994 to 2019. Information about each genome was collected from the BV-BRC database. Complete genome nucleotide sequences were downloaded from NCBI, while draft genome nucleotide sequences were downloaded from BV-BRC. SNPs from all genomes used in this study were extracted from Snippy core and used for alignment and maximum likelihood phylogenetic tree construction using RAxML-NG v. 1.1 (66). The sequences were aligned, and the resulting Newick tree was exported into Figtree v1.4.4 for display customization. Overlapping text and the genome information addition were edited with Inkscape v 1.2.2.

### SCC*mec* typing and antibiotic resistance genes

The SCC*mec* types were inferred from genomes using Staphopia-sccmec v 1.0.0 (67) with default settings. Staphopia-sccmec classifies SCC*mec* into 20 types (I–IX, *mecA* presence, subtypes Ia, IIa–b, IIIa, and IVa–d, g–h). The SCC*mec* typing results were reported using the most specific identification. For example, if a genome was classified as type I and type Ia, type Ia was used. The presence or absence of antimicrobial agent resistance-associated genes was determined using ABRicate (https://github.com/tseemann/abricate, Seemann ABRicate) with default settings. ABRicate made use of NCBI AMRFinderPlus (68).

## Data Availability

Raw reads and draft genomes for the sequenced isolates can be found under NCBI’s BioProject accession number PRJNA1089577 (https://www.ncbi.nlm.nih.gov/bioproject/PRJNA1089577) with raw reads being SRR28387494–SRR28387501, and draft genome DNA sequences are SAMN40545398–SAMN40545405. SRA links TD1: https://www.ncbi.nlm.nih.gov/sra/?term=SRR28387501 TD3: https://www.ncbi.nlm.nih.gov/sra/?term=SRR28387500 TD4: https://www.ncbi.nlm.nih.gov/sra/?term=SRR28387499 TD5: https://www.ncbi.nlm.nih.gov/sra/?term=SRR28387498 TD82: https://www.ncbi.nlm.nih.gov/sra/?term=SRR28387497 SP12: https://www.ncbi.nlm.nih.gov/sra/?term=SRR28387494 FA6: https://www.ncbi.nlm.nih.gov/sra/?term=SRR28387496 FA20: https://www.ncbi.nlm.nih.gov/sra/?term=SRR28387495 Biosample links TD1: https://www.ncbi.nlm.nih.gov/biosample/SAMN40545398 TD3: https://www.ncbi.nlm.nih.gov/biosample/SAMN40545399 TD4: https://www.ncbi.nlm.nih.gov/biosample/SAMN40545400 TD5: https://www.ncbi.nlm.nih.gov/biosample/SAMN40545401 TD82: https://www.ncbi.nlm.nih.gov/biosample/SAMN40545402 SP12: https://www.ncbi.nlm.nih.gov/biosample/SAMN40545405 FA6: https://www.ncbi.nlm.nih.gov/biosample/SAMN40545403 FA20: https://www.ncbi.nlm.nih.gov/biosample/SAMN40545404

## SUPPLEMENTAL MATERIAL

Table S1 spectrum.01995-24-s0001.xlsx Metadata available for genomes used in this study.

ASM does not own the copyrights to Supplemental Material that may be linked to, or accessed through, an article. The authors have granted ASM a non-exclusive, world-wide license to publish the Supplemental Material files. Please contact the corresponding author directly for reuse.

## References

[1] Davis JA, Jackson CR, Fedorka-Cray PJ, Barrett JB, Brousse JH, Gustafson J, Kucher M. 2014. Carriage of methicillin-resistant staphylococci by healthy companion animals in the US. Lett Appl Microbiol 59:1–8. doi:10.1111/lam.12254 24730724

[2] Penna B, Silva MB, Soares AER, Vasconcelos ATR, Ramundo MS, Ferreira FA, Silva-Carvalho MC, de Sousa VS, Rabello RF, Bandeira PT, de Souza VS, Planet PJ, Vieira-da-Motta O, Botelho AMN, Figueiredo AMS. 2021. Comparative genomics of MRSA strains from human and canine origins reveals similar virulence gene repertoire. Sci Rep 11:4724. doi:10.1038/s41598-021-83993-5 33633263 PMC7907190

[3] Becker K, Schaumburg F, Fegeler C, Friedrich AW, Köck R, Prevalence of Multiresistant Microorganisms PMM Study. 2017. Staphylococcus aureus from the German general population is highly diverse. Int J Med Microbiol 307:21–27. doi:10.1016/j.ijmm.2016.11.007 28017539

[4] Dalton KR, Ruble K, Redding LE, Morris DO, Mueller NT, Thorpe RJ, Agnew J, Carroll KC, Planet PJ, Rubenstein RC, Chen AR, Grice EA, Davis MF. 2021. Microbial sharing between pediatric patients and therapy dogs during hospital animal-assisted intervention programs. Microorganisms 9:1054. doi:10.3390/microorganisms9051054 34068292 PMC8153335

[5] Nienhoff U, Kadlec K, Chaberny IF, Verspohl J, Gerlach GF, Schwarz S, Simon D, Nolte I. 2009. Transmission of methicillin-resistant Staphylococcus aureus strains between humans and dogs: two case reports. J Antimicrob Chemother 64:660–662. doi:10.1093/jac/dkp243 19608580

[6] Manian FA. 2003. Asymptomatic nasal carriage of mupirocin-resistant, methicillin-resistant Staphylococcus aureus (MRSA) in a pet dog associated with MRSA infection in household contacts. Clin Infect Dis 36:e26–e28. doi:10.1086/344772 12522764

[7] Jevons MP. 1961. “Celbenin” - resistant Staphylococci. Br Med J 1:124–125. doi:10.1136/bmj.1.5219.124-a

[8] Hasanpour AH, Sepidarkish M, Mollalo A, Ardekani A, Almukhtar M, Mechaal A, Hosseini SR, Bayani M, Javanian M, Rostami A. 2023. The global prevalence of methicillin-resistant Staphylococcus aureus colonization in residents of elderly care centers: a systematic review and meta-analysis. Antimicrob Resist Infect Control 12:4. doi:10.1186/s13756-023-01210-6 36709300 PMC9884412

[9] Aires-de-Sousa M. 2017. Methicillin-resistant Staphylococcus aureus among animals: current overview. Clin Microbiol Infect 23:373–380. doi:10.1016/j.cmi.2016.11.002 27851997

[10] Lee AS, de Lencastre H, Garau J, Kluytmans J, Malhotra-Kumar S, Peschel A, Harbarth S. 2018. Methicillin-resistant Staphylococcus aureus. Nat Rev Dis Primers 4:18033. doi:10.1038/nrdp.2018.33 29849094

[11] Wertheim HFL, Melles DC, Vos MC, van Leeuwen W, van Belkum A, Verbrugh HA, Nouwen JL. 2005. The role of nasal carriage in Staphylococcus aureus infections. Lancet Infect Dis 5:751–762. doi:10.1016/S1473-3099(05)70295-4 16310147

[12] Gorwitz RJ, Kruszon-Moran D, McAllister SK, McQuillan G, McDougal LK, Fosheim GE, Jensen BJ, Killgore G, Tenover FC, Kuehnert MJ. 2008. Changes in the prevalence of nasal colonization with Staphylococcus aureus in the United States, 2001-2004. J Infect Dis 197:1226–1234. doi:10.1086/533494 18422434

[13] Boost MV, O’Donoghue MM, James A. 2008. Prevalence of Staphylococcus aureus carriage among dogs and their owners. Epidemiol Infect 136:953–964. doi:10.1017/S0950268807009326 17678561 PMC2870875

[14] Sun J, Yang M, Sreevatsan S, Davies PR. 2015. Prevalence and characterization of Staphylococcus aureus in growing pigs in the USA. PLoS One 10:e0143670. doi:10.1371/journal.pone.0143670 26599635 PMC4658009

[15] Virgin JE, Van Slyke TM, Lombard JE, Zadoks RN. 2009. Short communication: methicillin-resistant Staphylococcus aureus detection in US bulk tank milk. J Dairy Sci 92:4988–4991. doi:10.3168/jds.2009-2290 19762816

[16] Haran KP, Godden SM, Boxrud D, Jawahir S, Bender JB, Sreevatsan S. 2012. Prevalence and characterization of Staphylococcus aureus, including methicillin-resistant Staphylococcus aureus, isolated from bulk tank milk from Minnesota dairy farms. J Clin Microbiol 50:688–695. doi:10.1128/JCM.05214-11 22170937 PMC3295154

[17] Albrich WC, Harbarth S. 2008. Health-care workers: source, vector, or victim of MRSA? Lancet Infect Dis 8:289–301. doi:10.1016/S1473-3099(08)70097-5 18471774

[18] van den Broek IVF, van Cleef BAGL, Haenen A, Broens EM, van der Wolf PJ, van den Broek MJM, Huijsdens XW, Kluytmans JAJW, van de Giessen AW, Tiemersma EW. 2009. Methicillin-resistant Staphylococcus aureus in people living and working in pig farms. Epidemiol Infect 137:700–708. doi:10.1017/S0950268808001507 18947444

[19] Seguin JC, Walker RD, Caron JP, Kloos WE, George CG, Hollis RJ, Jones RN, Pfaller MA. 1999. Methicillin-resistant Staphylococcus aureus outbreak in a veterinary teaching hospital: potential human-to-animal transmission. J Clin Microbiol 37:1459–1463. doi:10.1128/JCM.37.5.1459-1463.1999 10203505 PMC84801

[20] Jordan D, Simon J, Fury S, Moss S, Giffard P, Maiwald M, Southwell P, Barton MD, Axon JE, Morris SG, Trott DJ. 2011. Carriage of methicillin-resistant Staphylococcus aureus by veterinarians in Australia. Aust Vet J 89:152–159. doi:10.1111/j.1751-0813.2011.00710.x 21495985

[21] Weese J, Dick H, Willey B, Mcgeer A, Kreiswirth B, Innis B, Low D. 2006. Suspected transmission of methicillin-resistant Staphylococcus aureus between domestic pets and humans in veterinary clinics and in the household. Vet Microbiol 115:148–155. doi:10.1016/j.vetmic.2006.01.004 16464540

[22] David MZ, Daum RS. 2010. Community-associated methicillin-resistant Staphylococcus aureus: epidemiology and clinical consequences of an emerging epidemic. Clin Microbiol Rev 23:616–687. doi:10.1128/CMR.00081-09 20610826 PMC2901661

[23] Pardos de la Gandara M, Curry M, Berger J, Burstein D, Della-Latta P, Kopetz V, Quale J, Spitzer E, Tan R, Urban C, Wang G, Whittier S, de Lencastre H, Tomasz A. 2016. MRSA causing infections in hospitals in Greater Metropolitan New York: major shift in the dominant clonal type between 1996 and 2014. PLoS One 11:e0156924. doi:10.1371/journal.pone.0156924 27272665 PMC4896443

[24] Lee BY, Singh A, David MZ, Bartsch SM, Slayton RB, Huang SS, Zimmer SM, Potter MA, Macal CM, Lauderdale DS, Miller LG, Daum RS. 2013. The economic burden of community-associated methicillin-resistant Staphylococcus aureus (CA-MRSA). Clin Microbiol Infect 19:528–536. doi:10.1111/j.1469-0691.2012.03914.x 22712729 PMC3463640

[25] Jensen JD, Christensen T, Olsen JV, Sandøe P. 2020. Costs and benefits of alternative strategies to control the spread of livestock-acquired methicillin-resistant Staphylococcus aureus from pig production. Value Health 23:89–95. doi:10.1016/j.jval.2019.07.006 31952677

[26] Zhang K, McClure JA, Elsayed S, Louie T, Conly JM. 2005. Novel multiplex PCR assay for characterization and concomitant subtyping of staphylococcal cassette chromosome mec types I to V in methicillin-resistant Staphylococcus aureus. J Clin Microbiol 43:5026–5033. doi:10.1128/JCM.43.10.5026-5033.2005 16207957 PMC1248471

[27] Foster T. 1996. Chapter 12 Staphylococcus. 4th ed. The University of Texas Medical Branch at Galveston, Galveston. https://www.ncbi.nlm.nih.gov/books/NBK8448/. 21413338

[28] Cosgrove SE. 2006. The relationship between antimicrobial resistance and patient outcomes: mortality, length of hospital stay, and health care costs. Clin Infect Dis 42:S82–S89. doi:10.1086/499406 16355321

[29] Hanselman BA, Kruth SA, Rousseau J, Weese JS. 2009. Coagulase positive staphylococcal colonization of humans and their household pets. Can Vet J 50:954–958. 19949556 PMC2726022

[30] Truong-Bolduc QC, Wang Y, Hooper DC. 2021. Staphylococcus aureus Tet38 efflux pump structural modeling and roles of essential residues in drug efflux and host cell internalization. Infect Immun 89:e00811-20. doi:10.1128/IAI.00811-20 33619028 PMC8091097

[31] Wang Y, Zhang P, Wu J, Chen S, Jin Y, Long J, Duan G, Yang H. 2023. Transmission of livestock-associated methicillin-resistant Staphylococcus aureus between animals, environment, and humans in the farm. Environ Sci Pollut Res 30:86521–86539. doi:10.1007/s11356-023-28532-7 37418185

[32] Fey PD, Saïd-Salim B, Rupp ME, Hinrichs SH, Boxrud DJ, Davis CC, Kreiswirth BN, Schlievert PM. 2003. Comparative molecular analysis of community- or hospital-acquired methicillin-resistant Staphylococcus aureus. Antimicrob Agents Chemother 47:196–203. doi:10.1128/AAC.47.1.196-203.2003 12499191 PMC149027

[33] Ma XX, Ito T, Tiensasitorn C, Jamklang M, Chongtrakool P, Boyle-Vavra S, Daum RS, Hiramatsu K. 2002. Novel type of staphylococcal cassette chromosome mec identified in community-acquired methicillin-resistant Staphylococcus aureus strains. Antimicrob Agents Chemother 46:1147–1152. doi:10.1128/AAC.46.4.1147-1152.2002 11897611 PMC127097

[34] Ito T, Katayama Y, Asada K, Mori N, Tsutsumimoto K, Tiensasitorn C, Hiramatsu K. 2001. Structural comparison of three types of staphylococcal cassette chromosome mec integrated in the chromosome in methicillin-resistant Staphylococcus aureus. Antimicrob Agents Chemother 45:1323–1336. doi:10.1128/AAC.45.5.1323-1336.2001 11302791 PMC90469

[35] Ito T, Ma XX, Takeuchi F, Okuma K, Yuzawa H, Hiramatsu K. 2004. Novel type V staphylococcal cassette chromosome mec driven by a novel cassette chromosome recombinase, ccrC. Antimicrob Agents Chemother 48:2637–2651. doi:10.1128/AAC.48.7.2637-2651.2004 15215121 PMC434217

[36] Bhutia KO, Singh TSK, Adhikari L, Biswas S. 2015. Molecular characterization of community- & hospital-acquired methicillin-resistant & methicillin-sensitive Staphylococcus aureus isolates in Sikkim. Indian J Med Res 142:330–335. doi:10.4103/0971-5916.166600 26458350 PMC4669869

[37] Gonzalez BE, Rueda AM, Shelburne SA 3rd, Musher DM, Hamill RJ, Hulten KG. 2006. Community-associated strains of methicillin-resistant Staphylococccus aureus as the cause of healthcare-associated infection. Infect Control Hosp Epidemiol 27:1051–1056. doi:10.1086/507923 17006811

[38] Schwaber MJ, Masarwa S, Navon-Venezia S, Kandlik Y, Chmelnitsky I, Smollan G, Glick R, Neria G, Carmeli Y. 2011. High prevalence of methicillin-resistant Staphylococcus aureus among residents and staff of long-term care facilities, involving joint and parallel evolution. Clin Infect Dis 53:910–913. doi:10.1093/cid/cir607 21984272

[39] Cotter CJ, Ferradas C, Ludwig S, Dalton K, Larsen J, Laucks D, Iverson SA, Baron P, Tolomeo PC, Brazil AM, Ferguson JM, Lautenbach E, Rankin SC, Morris DO, Davis MF. 2023. Risk factors for meticillin-resistant Staphylococcus aureus (MRSA) carriage in MRSA-exposed household pets. Vet Dermatol 34:22–27. doi:10.1111/vde.13135 36331035 PMC11168721

[40] Rutland BE, Weese JS, Bolin C, Au J, Malani AN. 2009. Human-to-dog transmission of methicillin-resistant Staphylococcus aureus. Emerg Infect Dis 15:1328–1330. doi:10.3201/eid1508.081635 19751611 PMC2815967

[41] Mork RL, Hogan PG, Muenks CE, Boyle MG, Thompson RM, Sullivan ML, Morelli JJ, Seigel J, Orscheln RC, Bubeck Wardenburg J, Gehlert SJ, Burnham CAD, Rzhetsky A, Fritz SA. 2020. Longitudinal, strain-specific Staphylococcus aureus introduction and transmission events in households of children with community-associated meticillin-resistant S aureus skin and soft tissue infection: a prospective cohort study. Lancet Infect Dis 20:188–198. doi:10.1016/S1473-3099(19)30570-5 31784369 PMC6995751

[42] Khairullah AR, Sudjarwo SA, Effendi MH, Ramandinianto SC, Gelolodo MA, Widodo A, Riwu KHP, Kurniawati DA. 2023. Pet animals as reservoirs for spreading methicillin-resistant Staphylococcus aureus to human health. J Adv Vet Anim Res 10:1–13. doi:10.5455/javar.2023.j641 37155545 PMC10122942

[43] Nagasundaram N, Sistla S. 2019. Existence of multiple SCCmec elements in clinical isolates of methicillin-resistant Staphylococcus aureus. J Med Microbiol 68:720–727. doi:10.1099/jmm.0.000977 30994438

[44] Katayama Y, Ito T, Hiramatsu K. 2001. Genetic organization of the chromosome region surrounding mecA in clinical staphylococcal strains: role of IS431-mediated mecI deletion in expression of resistance in mecA-carrying, low-level methicillin-resistant Staphylococcus haemolyticus. Antimicrob Agents Chemother 45:1955–1963. doi:10.1128/AAC.45.7.1955-1963.2001 11408208 PMC90585

[45] Ito T, Hiramatsu K, Oliveira DC, Lencastre H, Zhang K, Westh H, O’Brien F, Giffard PM, Coleman D, Tenover FC, Boyle-Vavra S, Skov RL, Enright MC, Kreiswirth B, Kwan SK, Grundmann H, Laurent F, Sollid JE, Kearns AM, Goering R, John JF, Daum R, Soderquist B. 2009. Classification of staphylococcal cassette chromosome mec (SCCmec): guidelines for reporting novel SCCmec elements. Antimicrob Agents Chemother 53:4961–4967. doi:10.1128/AAC.00579-09 19721075 PMC2786320

[46] Abdel-Moein KA, Zaher HM. 2019. Occurrence of multidrug-resistant methicillin-resistant Staphylococcus aureus among healthy farm animals: a public health concern. Int J Vet Sci Med 7:55–60. doi:10.1080/23144599.2019.1689630 31819891 PMC6882481

[47] Tomlin J, Pead MJ, Lloyd DH, Howell S, Hartmann F, Jackson HA, Muir P. 1999. Methicillin-resistant Staphylococcus aureus infections in 11 dogs. Vet Rec 144:60–64. doi:10.1136/vr.144.3.60 10070689

[48] Moses VK, Kandi V, Rao SKD. 2020. Minimum inhibitory concentrations of vancomycin and daptomycin against methicillin-resistant Staphylococcus aureus isolated from various clinical specimens: a study from south India. Cureus 12:e6749. doi:10.7759/cureus.6749 32140317 PMC7039364

[49] János D, Viorel H, Ionica I, Corina P, Tiana F, Roxana D. 2021. Carriage of multidrug resistance staphylococci in shelter dogs in Timisoara, Romania. Antibiotics (Basel) 10:801. doi:10.3390/antibiotics10070801 34356722 PMC8300769

[50] Moses IB, Santos FF, Gales AC. 2023. Human colonization and infection by Staphylococcus pseudintermedius: an emerging and underestimated zoonotic pathogen. Microorganisms 11:581. doi:10.3390/microorganisms11030581 36985155 PMC10057476

[51] Souza-Silva T, Rossi CC, Andrade-Oliveira AL, Vilar LC, Pereira MF, Penna B de A, Giambiagi-deMarval M. 2022. Interspecies transfer of plasmid-borne gentamicin resistance between Staphylococcus isolated from domestic dogs to Staphylococcus aureus. Infect Genet Evol 98:105230. doi:10.1016/j.meegid.2022.105230 35104683

[52] Frosini SM, Bond R, McCarthy AJ, Feudi C, Schwarz S, Lindsay JA, Loeffler A. 2020. Genes on the move: in vitro transduction of antimicrobial resistance genes between human and canine staphylococcal pathogens. Microorganisms 8:1–12. doi:10.3390/microorganisms8122031 PMC7766859 33353175

[53] Jolley KA, Bray JE, Maiden MCJ. 2018. Open-access bacterial population genomics: BIGSdb software, the PubMLST.org website and their applications. Wellcome Open Res 3:124. doi:10.12688/wellcomeopenres.14826.1 30345391 PMC6192448

[54] Enright MC, Day NPJ, Davies CE, Peacock SJ, Spratt BG. 2000. Multilocus sequence typing for characterization of methicillin-resistant and methicillin-susceptible clones of Staphylococcus aureus. J Clin Microbiol 38:1008–1015. doi:10.1128/JCM.38.3.1008-1015.2000 10698988 PMC86325

[55] Shakeri F, Shojai A, Golalipour M, Rahimi Alang S, Vaez H, Ghaemi EA. 2010. Spa diversity among MRSA and MSSA strains of Staphylococcus aureus in north of Iran. Int J Microbiol 2010:351397. doi:10.1155/2010/351397 20862383 PMC2939385

[56] Johnston A, Olsen S, Sreevatsan S, Bender J. Methicillin-resistant Staphylococcus aureus isolated from therapy dogs and handlers within a hospital setting. University of Minnesota Poster Presentation.

[57] Wood DE, Lu J, Langmead B. 2019. Improved metagenomic analysis with Kraken 2. Genome Biol 20:257. doi:10.1186/s13059-019-1891-0 31779668 PMC6883579

[58] Martin M. 2011. Cutadapt removes adapter sequences from high-throughput sequencing reads. EMBnet j 17:10. doi:10.14806/ej.17.1.200

[59] Alonge M, Soyk S, Ramakrishnan S, Wang X, Goodwin S, Sedlazeck FJ, Lippman ZB, Schatz MC. 2019. RaGOO: fast and accurate reference-guided scaffolding of draft genomes. Genome Biol 20:224. doi:10.1186/s13059-019-1829-6 31661016 PMC6816165

[60] Alonge M, Lebeigle L, Kirsche M, Jenike K, Ou S, Aganezov S, Wang X, Lippman ZB, Schatz MC, Soyk S. 2022. Automated assembly scaffolding using RagTag elevates a new tomato system for high-throughput genome editing. Genome Biol 23:258. doi:10.1186/s13059-022-02823-7 36522651 PMC9753292

[61] Golding GR, Levett PN, McDonald RR, Irvine J, Quinn B, Nsungu M, Woods S, Khan M, Ofner-Agostini M, Mulvey MR, Northern Antibiotic Resistance Partnership. 2011. High rates of Staphylococcus aureus USA400 infection, Northern Canada. Emerg Infect Dis 17:722–725. doi:10.3201/eid1704.100482 21470471 PMC3377391

[62] Vignaroli C, Varaldo PE, Camporese A. 2009. Methicillin-resistant Staphylococcus aureus USA400 Clone, Italy. Emerg Infect Dis 15:995–996. doi:10.3201/eid1506.081632 19523322 PMC2727323

[63] Grant JR, Enns E, Marinier E, Mandal A, Herman EK, Chen C-Y, Graham M, Van Domselaar G, Stothard P. 2023. Proksee: in-depth characterization and visualization of bacterial genomes. Nucleic Acids Res 51:W484–W492. doi:10.1093/nar/gkad326 37140037 PMC10320063

[64] Seemann T. 2014. Prokka: rapid prokaryotic genome annotation. Bioinformatics 30:2068–2069. doi:10.1093/bioinformatics/btu153 24642063

[65] Camacho C, Coulouris G, Avagyan V, Ma N, Papadopoulos J, Bealer K, Madden TL. 2009. BLAST+: architecture and applications. BMC Bioinformatics 10:421. doi:10.1186/1471-2105-10-421 20003500 PMC2803857

[66] Kozlov AM, Darriba D, Flouri T, Morel B, Stamatakis A. 2019. RAxML-NG: a fast, scalable and user-friendly tool for maximum likelihood phylogenetic inference. Bioinformatics 35:4453–4455. doi:10.1093/bioinformatics/btz305 31070718 PMC6821337

[67] Petit RA, Read TD. 2018. Staphylococcus aureus viewed from the perspective of 40,000+ genomes. PeerJ 6:e5261. doi:10.7717/peerj.5261 30013858 PMC6046195

[68] Feldgarden M, Brover V, Gonzalez-Escalona N, Frye JG, Haendiges J, Haft DH, Hoffmann M, Pettengill JB, Prasad AB, Tillman GE, Tyson GH, Klimke W. 2021. AMRFinderPlus and the Reference Gene Catalog facilitate examination of the genomic links among antimicrobial resistance, stress response, and virulence. Sci Rep 11:12728. doi:10.1038/s41598-021-91456-0 34135355 PMC8208984

